# Editorial to “A critical analysis of online patient‐directed resources on catheter ablation for ventricular arrhythmias”

**DOI:** 10.1002/joa3.70046

**Published:** 2025-03-24

**Authors:** Reina Tonegawa‐Kuji

**Affiliations:** ^1^ Department of Medical and Health Information Management National Cerebral and Cardiovascular Center Suita Japan

Catheter ablation is a critical treatment for managing life‐threatening ventricular arrhythmias. To make informed decisions, patients need clear and accurate information about the procedure, its risks, and alternative treatment options. While healthcare providers explain these aspects during consultations, catheter ablation is a complex procedure, making it difficult for patients to fully understand everything in a single discussion. As a result, many turn to online resources for additional information. Reliable online information is crucial in helping patients navigate their treatment choices. In a recent study, “A Critical Analysis of Online Patient‐Directed Resources on Catheter Ablation for Ventricular Arrhythmias,” Sood A et.al. evaluated the readability and quality of online patient materials.^1^ The findings showed that among the 60 resources analyzed, not a single one met both the ideal readability and quality standards. Specifically, none of the reviewed resources met the readability standard recommended by the American Medical Association (AMA), which advises that patient‐directed materials be written at a 6th‐grade reading level or lower.^2^ Furthermore, only one resource was written at or below the average American literacy level of 8th grade.^3^ Additionally, only 27% of the resources scored as high quality based on the JAMA score.^4^ The study also found no correlation between readability and quality. Beyond written materials, the study also examined videos as an alternative medium for patient education. While videos can be an effective tool for improving comprehension, the study found that only five (21%) of the 24 online videos included all the essential elements necessary for informed consent—clear explanations of the procedure, its risks, and available alternatives. This highlights a broader issue: not only are written resources too complex, but even multimedia formats fail to provide complete, accessible information.

There are several reasons why online medical information may not be tailored to patients' level of understanding. One key factor is the significant knowledge gap between healthcare providers and patients. When medical professionals attempt to explain procedures or treatments, they may unintentionally use overly complex language, simply because they do not realize how difficult these terms are for non‐experts to understand. Additionally, many healthcare professionals are unaware that patient‐directed materials should be written at a 6th‐grade reading level or lower. When creating educational resources, it is common to simplify literature originally intended for medical professionals, such as guidelines from academic organizations. However, to make these materials truly accessible, greater emphasis must be placed on readability, ensuring that content is clear and easy for patients to understand.

Addressing this issue requires a more patient‐centered approach. One effective strategy is incorporating feedback from individuals who have undergone catheter ablation. Their insights can help shape materials that align better with patient needs. Additionally, visual aids such as videos and diagrams can enhance comprehension, but they must go beyond procedural explanations to include essential informed consent topics, such as risks and alternative treatment options. Once high‐quality, patient‐friendly materials are developed, a system should be in place to make them widely available to all healthcare institutions. Standardizing and sharing these resources would ensure that patients, regardless of where they seek care, receive consistent and reliable information. To address this issue, the authors of the study developed an example of a patient‐friendly document on ventricular arrhythmia catheter ablation, written at the AMA‐recommended 6th‐grade level. Available in the study's appendix, this serves as a model for making complex medical information more accessible.

Moving forward, greater efforts are needed to create materials that truly empower patients rather than overwhelm them with complexity. This study has shed light on that need, providing a strong foundation for future improvements in patient education. Ensuring that all patients, regardless of literacy level, have access to clear, accurate, and comprehensive medical information is not just a recommendation—it is necessity for informed decision‐making and better health outcomes.
